# Whole Genome Characterization of *Leptospira kirschneri* Serogroup Pomona in Croatia: Insights into Its Diversity and Evolutionary Emergence

**DOI:** 10.3390/pathogens14090860

**Published:** 2025-08-29

**Authors:** Iva Benvin, Taylor K. Paisie, Ines Caetano Varanda, Zachary P. Weiner, Robyn A. Stoddard, Jay E. Gee, Christopher A. Gulvik, Chung K. Marston, Vesna Mojčec Perko, Zrinka Štritof, Josipa Habuš, Josip Margaletić, Marko Vucelja, Linda Bjedov, Nenad Turk

**Affiliations:** 1 Department of Microbiology and Infectious Diseases with Clinic, Faculty of Veterinary Medicine, University of Zagreb, 10000 Zagreb, Croatia; vmojcec@vef.unizg.hr (V.M.P.); zstritof@vef.unizg.hr (Z.Š.); jhabus@vef.unizg.hr (J.H.); turk@vef.unizg.hr (N.T.); 2 Centers for Disease Control and Prevention, National Center for Emerging and Zoonotic Infectious Diseases, Division of High-Consequence Pathogens and Pathology, Bacterial Special Pathogens Branch, Atlanta 30333, Georgia; xxd7@cdc.gov (Z.P.W.); frd8@cdc.gov (R.A.S.); xzg4@cdc.gov (J.E.G.); cdk5@cdc.gov (C.K.M.); 3 National Institute of Agricultural and Veterinary Research, IP (INIAV), 2780-157 Lisbon, Portugal; ines.caetano@iniav.pt; 4 Faculty of Forestry and Wood Technology, University of Zagreb, 10000 Zagreb, Croatia; jmargaletic@sumfak.unizg.hr (J.M.); mvucelja@sumfak.unizg.hr (M.V.); lbjedov@sumfak.unizg.hr (L.B.)

**Keywords:** *Leptospira kirschneri*, serogroup Pomona, whole genome sequencing, phylogenetic analysis, small rodents, One Health

## Abstract

Leptospirosis is a worldwide zoonosis caused by pathogenic *Leptospira* spp. with small rodents serving as the main reservoir. In Croatia, the serogroup Pomona has been detected most frequently, but its genomic diversity remains insufficiently characterized. This study presents the first whole genome sequencing analysis of 48 Croatian *Leptospira* spp. isolates collected from small rodents over a 14-year period. Serological typing confirmed that all the isolates belonged to the serogroup Pomona. Genomic analysis assigned them to *L. kirschneri* based on high genomic similarity using average nucleotide identity (ANI). The isolates were assigned to ST-98 using traditional multilocus sequence typing (MLST), while cgMLST identified seven genotype clusters, many of which showed geographic structuring. Phylogenetic analyses based on single nucleotide polymorphisms (SNPs) supported this structure and revealed a monophyletic clade of Croatian isolates distinct from other global *L. kirschneri* strains. Serological typing, MLST, and phylogenetic clustering support classification of the isolates as *L. kirschneri*, serogroup Pomona, most likely serovar Mozdok, although serovar Tsaratsovo cannot be excluded. These results indicate the existence of a geographically restricted and potentially host-adapted lineage of *L. kirschneri* in Croatia. The integration of ecological, serological, and genomic data in this study emphasizes the value of whole genome sequencing for understanding the population biology of *Leptospira* spp. serogroup Pomona. Moreover, it supports targeted, country-specific surveillance and control strategies for leptospirosis through the identification of circulating serovars and species in reservoir hosts, in line with a One Health approach.

## 1. Introduction

Leptospirosis is a globally distributed, re-emerging infectious disease caused by pathogenic bacteria of the genus *Leptospira* [1]. It affects a wide range of domestic and wild animals as well as humans, posing significant public health concerns [2]. Leptospires are immunologically and genetically heterogeneous microorganisms. The genus *Leptospira* is currently classified into 69 species grouped into four subclades, with more than 300 serovars of pathogenic *Leptospira* organized into 30 serogroups [2,3]. Differences in the pathogenicity of the species and serovars, the susceptibility and immune response of the hosts, and the infectious dose lead to different clinical manifestations ranging from mild or subclinical infections to severe, life-threatening outcomes [1,4].

Small rodents serve as the main reservoirs of *Leptospira* spp. and play a crucial role in the persistence of the disease in the environment. Once infected, they become asymptomatic carriers that shed leptospires in their urine continuously or intermittently throughout their lifetime, serving as an important source of infection [1,4]. In humans and animals, infection occurs through mucous membranes and microlesions of the skin via infected urine or contaminated water or soil [5,6]. Following the bacteriemic stage, the leptospires colonize the renal proximal tubular epithelial cells and are excreted in the urine [4,6], contaminating the environment, where pathogenicity may be retained for up to 20 months [7,8].

Hemolysin-producing serogroups such as Pomona or Icterohaemorrhagiae are commonly associated with pronounced clinical symptoms in both animals and humans [4,9]. Among them, the serogroup Pomona is of particular importance due to its increasing trend of occurrence in various domestic and wild animal species worldwide [10,11,12,13,14,15,16]. Moreover, over the last decade, research has identified the serogroup Pomona as the most prevalent serogroup in various hosts in Croatia [17,18,19]. The serogroup Pomona consists of eight serovars—Altodouro, Kennewicki, Kunming, Mozdok, Pomona, Proechimys, Tropica, and Tsaratsovo—which are distributed across five distinct species: *L. interrogans*, *L. kirschneri*, *L. borgpetersenii*, *L. noguchii*, and *L. santarosai* [20,21,22,23,24,25,26]. Pigs have been considered the main carriers of the serogroup Pomona, particularly for the serovar Pomona [27], whereas black-striped field mice (*Apodemus agrarius*) are the primary reservoirs for the serovar Mozdok [28].

The complex taxonomy of the genus *Leptospira* makes diagnosis challenging. Most laboratory diagnoses and epizootiologic/epidemiologic studies rely on serologic and molecular methods [29,30,31]. The microscopic agglutination test (MAT) identifies the presumptive infectious serogroup but not the specific serovar [32], while molecular techniques can determine the *Leptospira* spp. species, but not the infecting serogroup or serovar [33]. Sequencing PCR products from positive clinical samples is feasible but often hampered by the low abundance of leptospiral DNA, which can compromise sequencing quality. Furthermore, the successful isolation of *Leptospira* spp. in media enables the use of advanced molecular techniques such as whole genome sequencing (WGS) to identify and thoroughly characterize specific strains. WGS enables the analysis of genomic variation, which is particularly valuable for comparing strains from geographically or ecologically diverse regions [34].

In Croatia, leptospirosis is an endemic disease and a significant veterinary and public health concern. The role of small rodents in the epizootiologic and epidemiologic cycle of leptospirosis in Croatia has been studied over the years [17,28,35,36,37]. These rodents enable the long-term survival and persistence of *Leptospira* spp. in the environment, and characterization of the strains they harbor provides insight into the currently circulating pathogenic *Leptospira* species and serovars.

The aim of this study is to analyze the whole genomes of *Leptospira* spp. strains belonging to the serogroup Pomona, isolated over time from small rodents, to characterize their genomic features and diversity. A deeper understanding of the genomic characteristics of the *Leptospira* spp. serogroup Pomona should provide valuable insights into its pathogenic potential, epidemiology, and evolutionary emergence.

## 2. Materials and Methods

### 2.1. Investigated Leptospira spp. Isolates

A total of 48 archived isolates of *Leptospira* spp. isolated from the kidneys of small rodents were used for this study. These isolates were collected over a 14-year period from various regions of Croatia, with available data on the small rodent species, collection location, and date of sampling. The small rodent species *Apodemus agrarius* and *Microtus lavernedii* were identified on the basis of morphological characteristics, while *Apodemus flavicollis* and *Apodemus sylvaticus*, which are morphologically indistinguishable, were differentiated using polymerase chain reaction (PCR) targeting the mitochondrial *cytochrome b* gene, followed by sequencing of the PCR products. The cultures were part of the collection of pathogenic leptospires in the Laboratory of Leptospires at the Faculty of Veterinary Medicine, University of Zagreb, where they were maintained in Korthof and Fletcher media.

### 2.2. Serological Typing of Leptospira spp. Isolates

The affiliation of the analyzed isolates of *Leptospira* spp. to specific serogroups was tested using a panel of 14 reference hyperimmune sera (Table S1) prepared in rabbits (OIE Reference Laboratory for Leptospirosis, AMC, Amsterdam, The Netherlands) according to a standard procedure [38]. *Leptospira* spp. cultures cultivated in Korthof medium for up to 10 days, having a density of 2–4 × 10^8^ bacteria/mL, were used as antigens. Serial dilutions of the tested sera were prepared in microtiter plates with phosphate-buffered saline (PBS), starting with a dilution of 1:50. After a 2-hour incubation at 28–30 °C, the results were read under a darkfield microscope. A serologically positive reaction was determined by the presence of agglutinated leptospires compared to the negative control. The endpoint titer was the highest serum dilution showing at least 50% agglutination of the leptospires. The infectious serogroup of the culture was determined based on the hyperimmune serum showing the highest agglutination titer.

### 2.3. Genomic Characterization of Leptospira spp. Strains

#### 2.3.1. DNA Extraction

*Leptospira* spp. cultures up to 10 days old having a density of 2–4 × 10^8^ bacteria/mL in Korthof media were sent to the Zoonoses and Select Agent Laboratory, Centers for Disease Control and Prevention, Atlanta, GA, USA, where further analyses and processing were conducted to identify, characterize, and study the strains.

Pretreatment of the *Leptospira* spp. cultures for DNA extraction included centrifugation at 4000 rpm for 15 min, followed by removal of the supernatant. The resulting pellets were then resuspended in 400 µL PBS. DNA extraction was subsequently performed using the Maxwell CSC 48 automated extraction system (Promega Corporation, Madison, WI, USA) with the Maxwell^®^ RSC Cultured Cells DNA Kit (Promega Corporation, Madison, WI, USA).

#### 2.3.2. Whole Genome Sequencing (WGS)

Strains isolated from the kidneys of small rodents determined with serological typing and belonging to the *Leptospira* spp. serogroup Pomona were analyzed by whole genome sequencing. The workflow for whole genome sequencing included DNA extraction (minimum concentration of 3.3 ng/µL and purity of A260/A280 between 1.8 and 2.0) and library preparation using the Nextera XT DNA Library Preparation Kit (Illumina, San Diego, CA, USA). Sequencing was performed on the Illumina MiSeq platform (Illumina, San Diego, CA, USA) using the MiSeq v3 600 cycle kit (2 × 300 bp reads) (Illumina, San Diego, CA, USA).

#### 2.3.3. Genome Assembly

Genome assembly of the sequenced samples was conducted using a Nextflow v24.04.2 assembly pipeline with default parameters developed by the bioinformatics team at the Zoonoses and Select Agent Laboratory (ZSAL) of the Centers for Disease Control and Prevention (CDC) in Atlanta, Georgia, USA (https://github.com/bacterial-genomics/wf-paired-end-illumina-assembly, v3.0.0). This pathogen-agnostic, general-purpose workflow is specifically designed for genome assembly of Illumina paired-end sequence data and was employed to ensure accurate and efficient data assembly. To improve assembly quality, the assemblies were filtered to remove contigs shorter than 500 bp, and all genomes retained had a minimum sequencing coverage of 39.5× (median 53.3×, range 39.5×–74.4×). These quality control steps ensured that only high-quality assemblies were used for the downstream analyses.

#### 2.3.4. Genomic Analyses

##### Average Nucleotide Identity (ANI)

To assess genomic relatedness among isolates, average nucleotide identity (ANI) was computed using the automated workflow available at the bacterial-genomics GitHub repository (https://github.com/bacterial-genomics/wf-ani, v1.0.0). The workflow implements three different ANI calculation methods—Biopython v1.6.8 [39], FastANI v1.33 (ParBLiSS/FastANI: Fast Whole-Genome Similarity (ANI) Estimation), and SKANI v0.1.3 [40]—providing comprehensive and robust pairwise identity metrics for genome comparisons.

**Figure 1:**
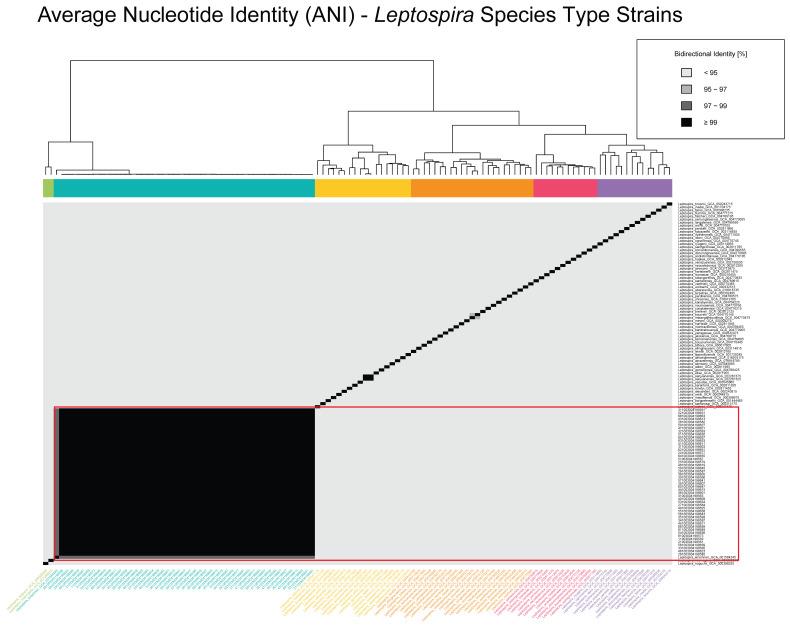
Average nucleotide identity (ANI) heatmap of *Leptospira* species type strains. Pairwise bidirectional ANI values are shown for representative genomes across diverse *Leptospira* species, including Croatian *L. kirschneri* isolates and reference type strains obtained from NCBI RefSeq (Table S4). The heatmap is color-coded by percent identity, with darker shades indicating higher similarity: <95% (lightest), 95–97%, 97–99%, and ≥99% (darkest). The Croatian isolates form a distinct cluster having ≥99% identity, consistent with high intra-lineage similarity. Dendrograms adjacent to the heatmap depict hierarchical clustering of genomes based on pairwise ANI values, and side bars represent clustering patterns across the dataset.

**Figure 2:**
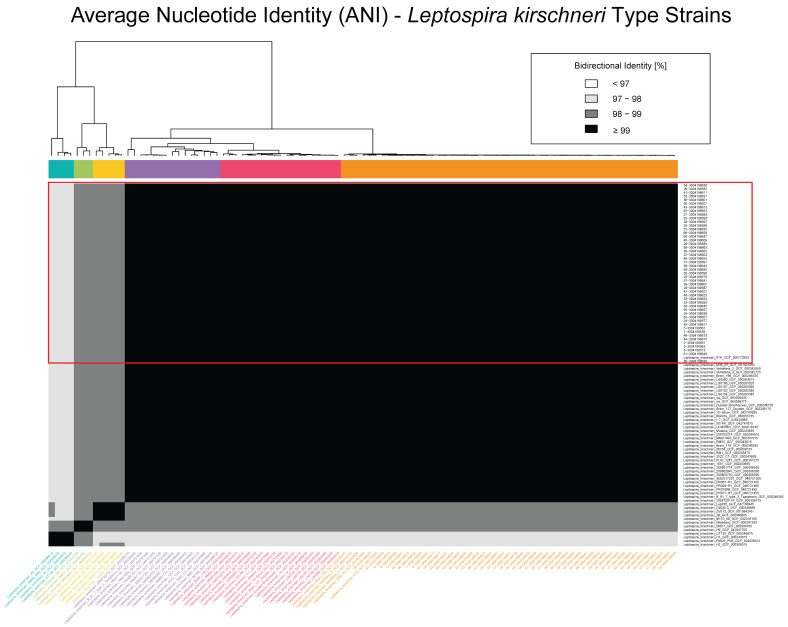
Average nucleotide identity (ANI) heatmap of *Leptospira kirschneri* genomes. Pairwise ANI values are shown for 99 *L. kirschneri* genomes, including 48 isolates from Croatia and 51 publicly available genomes from the NCBI RefSeq database (Table S5). The heatmap is color-coded by percent identity, with darker shades representing higher similarity: <95% (lightest), 95–97%, 97–99%, and ≥99% (darkest). The Croatian isolates form a tightly clustered group having ≥99% identity, consistent with high intra-lineage similarity, but exhibit lower identity values (95–98%) when compared to other *L. kirschneri* genomes, highlighting the genetic distinctiveness of the Croatian lineage. The dendrogram adjacent to the matrix represents hierarchical clustering based on pairwise ANI values, and colored side bars indicate the resulting genomic clusters.

In total, 118 genomes are included in Figure 1, with 70 representing recognized *Leptospira* spp. downloaded from the NCBI RefSeq database (RefSeq: NCBI Reference Sequence Database) as species type strains and the 48 sequenced *Leptospira* spp. isolates obtained from rodent populations sampled across locations in Croatia. Another dataset having a total of 99 genomes was also constructed, using the 48 *Leptospira* spp. samples collected in Croatia and 51 *L. kirschneri* isolates downloaded from NCBI’s RefSeq database (RefSeq: NCBI Reference Sequence Database) (Figure 2).

The analysis was performed using the Nextflow workflow manager [41], facilitating reproducibility and scalability. Briefly, the workflow involved the following key steps: preparation of genome input files in FASTA format; all pairwise ANI calculations among reference and Croatian isolate genomes, using Biopython, FastANI, and SKANI. Default parameters were employed, and ANI values above 95% were used as the threshold to define genomic species boundaries, consistent with accepted standards for prokaryotic species delineation.

##### Pangenome Analysis

A pangenome analysis was performed on the 48 Croatian *Leptospira* spp. genome assemblies to characterize core and accessory genome components. Prior to annotation, the assemblies were filtered to remove contigs shorter than 500 bp to minimize low-quality sequences. Genome annotation was then conducted using Bakta v1.9.4, a rapid prokaryotic genome annotation tool that provides standardized GFF3 output files [42]. The annotated GFF3 files were subsequently processed using Panaroo v1.3.4 [43], a robust pipeline for pangenome analysis that accounts for gene presence–absence variation and sequence fragmentation. Panaroo was run in strict mode with a 95% sequence identity threshold for gene clustering to minimize false positives and correct for assembly or annotation errors, while all the other parameters were left at their default values. The resulting output included a gene presence–absence matrix and definitions of core and accessory genome components. Gene presence–absence patterns were visualized using R v4.3.1 with the packages ggplot2 v3.5.1 and dplyr v1.1.4 to explore genomic diversity and to identify genes associated with specific clusters or traits. The pangenome was further analyzed to estimate the size of the core genome and the total gene repertoire across all the isolates.

##### Core Genome Multilocus Sequence Typing (cgMLST)

A gene-by-gene approach was used to define a core genome multilocus sequence typing (cgMLST) scheme using chewBBACA v3.3.10 [44]. Coding sequences (CDSs) were predicted for the 48 Croatian *Leptospira* spp. genome assemblies using Prodigal v2.6.3, followed by clustering based on BLAST Score Ratio (BSR) analysis to retain non-paralogous, high-quality loci [45]. The default BSR threshold of 0.6 implemented in chewBBACA was applied to filter paralogous loci. Incomplete loci were further excluded by requiring presence in at least 95% of genomes, forming the final cgMLST schema. A preliminary cgMLST schema for *Leptospira* was downloaded from BIGSdb v1.51.4 [46] as the basis for locus selection.

Allele calling was performed on the same 48 assemblies using the curated schema, generating allelic profiles and identifying novel alleles. The resulting allele table was used to extract the core genome loci and calculate presence–absence matrices.

The final cgMLST schema and allele profiles were integrated into a local BIGSdb v1.51.4 instance [46]. The locus definitions and allele sequences were uploaded through the schema definition interface, and the allelic profiles were used to assign core genome sequence types (cgSTs). This framework facilitated querying, clustering, and comparative analysis of allelic diversity across the Croatian isolates.

##### Whole Genome Single Nucleotide Polymorphism (SNP) Analysis

To investigate the genomic variation among the isolates, the wf-assembly-snps pipeline (https://github.com/bacterial-genomics/wf-assembly-snps, v1.0.3) was applied, a reproducible workflow built using Nextflow v24.04.2 [41]. This pipeline performs reference-free SNP calling and phylogenetic reconstruction from whole genome assemblies using Parsnp v1.5.6 [47].

The analysis was conducted in two phases. First, the pipeline was run on the 48 Croatian *Leptospira* spp. assembled genomes. Second, the same pipeline was executed using an expanded dataset that included the 48 Croatian genomes along with 23 additional *Leptospira* spp. assemblies obtained from NCBI RefSeq. In both runs, draft assemblies in FASTA format were used as the input.

Parsnp was used to align the core genome regions of the assemblies, identify high-confidence SNPs, and generate a multiple sequence alignment. The SNP alignment was used for phylogenetic tree creation with the program IQ-TREE v2.4.0 [48].

Phylogenetic trees and SNP alignments were further analyzed and visualized using R packages such as ape v5.8.1 [49] and ggtree v3.10.0 [50] to assess evolutionary relationships and genetic clustering among the isolates.

#### 2.3.5. Phylogenetic Analysis

A maximum likelihood phylogeny was inferred using IQ-TREE v2.4.0 [48] from the core SNP alignment generated from the 48 *Leptospira* spp. isolates collected in Croatia and 23 additional *L. kirschneri* genomes obtained from the NCBI RefSeq database. The best-fitting substitution model was determined automatically using ModelFinder based on the Bayesian Information Criterion. Branch support was assessed using 1000 ultrafast bootstrap replicates [48].

The resulting phylogenetic tree was visualized and annotated in R using the ggtree package v3.10.0 [50]. Tree topology and bootstrap values were used to assess clustering patterns, evolutionary relationships, and potential geographic or host-associated structure among the isolates.

#### 2.3.6. Computational Resources

Analyses were conducted on a high-performance computing cluster. Workflow execution was containerized using Docker to maintain consistency and reproducibility across all the steps. All the scripts and data processing procedures are publicly accessible on GitHub, promoting transparency and reproducibility.

## 3. Results

### 3.1. Leptospira spp. Isolates and Serological Typing

Detailed information on the individual isolates, including rodent species, sampling locations and dates, is provided in the Supplementary Materials (Table S2). Rodent species identification revealed that 42 isolates were from *Apodemus agrarius*, 5 from *Apodemus flavicollis* and 1 from *Microtus lavernedii*. All 48 isolates of *Leptospira* spp. tested with a panel of 14 hyperimmune reference sera showed agglutination exclusively with the serogroup Pomona, with titers ≥ 3200, clearly indicating a significantly higher reactivity to Pomona and confirming that all the isolates belonged to this serogroup.

### 3.2. Genome Assembly Quality and Metrics

High-quality whole genome sequencing data were obtained for all 48 *Leptospira* spp. isolates collected in Croatia (Table S3). Depth of coverage ranged from 39.5× to 74.4× (median: 53.3×), providing strong support for accurate de novo assembly. The assemblies consisted of between 50 and 95 contigs (median: 58), with total genome lengths spanning 4,389,636 bp to 4,428,928 bp (median: 4,413,607 bp), consistent with the expected size range for *L. kirschneri*. N50 values, reflecting assembly contiguity, ranged from 82,966 bp to 218,205 bp (median: 175,746 bp). GC content was highly uniform across all the samples, having a mean of 35.87% ± 0.004%, aligning with published values for this species. Collectively, these metrics demonstrate the high quality and consistency of the assemblies, supporting their use in comparative genomics and phylogenetic analyses.

### 3.3. Average Nucleotide Identity Confirms Species Delineation and Intraspecific Diversity

The ANI analysis was used to assess the genomic relatedness of the 48 *Leptospira* spp. isolates from Croatia in the context of both inter- and intraspecies diversity (Figure 1 and Figure 2). Figure 1 presents the ANI values for a dataset of 118 genomes, which includes the 48 Croatian *Leptospira* spp. isolates and 70 publicly available genomes representing all recognized *Leptospira* spp. type strains with available whole genome sequences from the NCBI RefSeq database (Table S4). The heatmap revealed a distinct genomic cluster containing all the Croatian isolates, with bidirectional ANI values exceeding 95% in comparisons with the *L. kirschneri* reference genome, confirming their species-level classification. These isolates also formed a discrete clade, clearly separated from other *Leptospira* species, which exhibited ANI values below the 95% threshold, reaffirming species-level divergence.

Figure 2 focuses on a higher-resolution comparison among 99 *L. kirschneri* genomes, including the 48 Croatian isolates and 51 additional *L. kirschneri* genomes from the NCBI RefSeq database (Table S5). Within this subset, the Croatian isolates formed a tightly clustered group with pairwise ANI values consistently exceeding 99%, indicative of a highly clonal population. Several additional subclusters were observed among non-Croatian isolates, with ANI values ranging from 97% to 99%, reflecting a broader spectrum of genomic diversity within *L. kirschneri*. Notably, despite their close identity, the Croatian isolates remained genetically distinct from all the other global reference strains, reinforcing their phylogeographic cohesion.

This pattern was further supported by pangenome analysis (Figure S1), which revealed that the Croatian isolates shared a large and stable core genome, consistent with clonal structure. While accessory gene variation was detected, it was relatively limited and showed no clear association with sampling location or other metadata, underscoring the genetic uniformity of this regional lineage.

Overall, the ANI analysis supports the assignment of all the Croatian isolates to *L. kirschneri* and highlights their high genomic similarity, while also contextualizing them within the broader diversity of the genus and species. These results corroborate phylogenetic and MLST-based findings and further demonstrate the utility of ANI for high-resolution bacterial population genomics.

### 3.4. Multilocus Sequence Typing (MLST) and Core Genome MLST (cgMLST)

**Figure 3:**
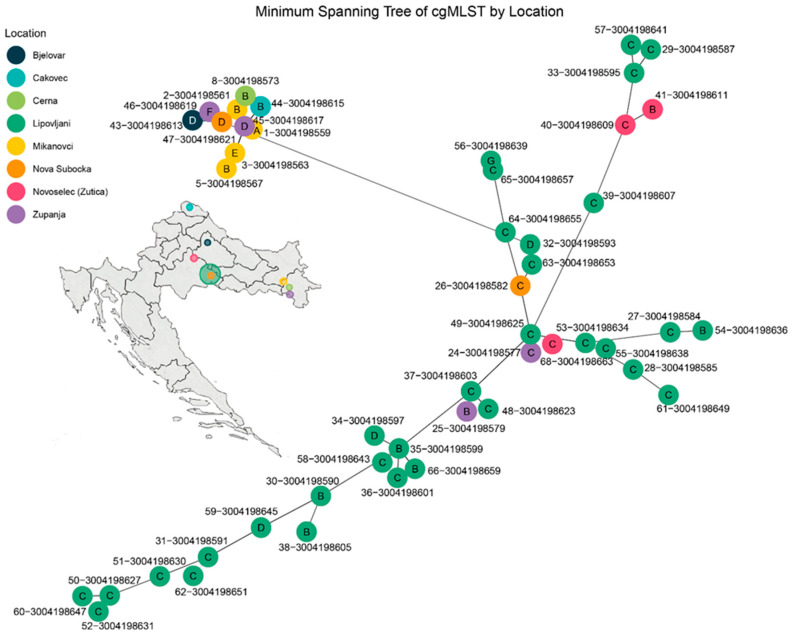
Minimum spanning tree (MST) of *Leptospira kirschneri* isolates based on core genome multilocus sequence typing (cgMLST). The MST was constructed from allele profiles of 48 isolates collected from rodents across eight locations in Croatia. Each node represents a single isolate and is color-coded by sampling location. Genotype clusters (A–G) are labeled within the nodes and were defined based on allelic similarity and MST topology. Edges represent the smallest number of allelic mismatches between profiles. Genotype C (cgST-1017) occupies the central portion of the network. Genotype B includes cgSTs 1012, 1016, and 1017; Genotype D includes cgSTs 1016 and 1017; Genotype E corresponds to cgST-1012; Genotype F includes cgSTs 959 and 1017; and Genotype G includes cgSTs 1011, 1013, and 1849.

All 48 *Leptospira* isolates from Croatia were assigned to sequence type ST-98 according to MLST scheme 3, indicating a highly clonal population structure at the seven-locus level (Table S6). This sequence type is affiliated with the serogroup Pomona and the genomic species *L. kirschneri* and is consistent with the serovars Mozdok or Tsaratsovo. Core genome MLST (cgMLST) analysis resolved the isolates into seven genotype clusters (designated A–G) based on allelic similarity and minimum spanning tree topology (Figure 3). These clusters corresponded to the following cgSTs: A (cgST-1016), B (cgST-1012, 1016, 1017), C (cgST-1017), D (cgST-1016, 1017), E (cgST-1012), F (cgST-959, 1017), and G (cgST-1011, 1013, 1849) (Table S3).

Distinct geographic patterns were observed among the genotype clusters. Genotype C exhibited strong spatial clustering, predominating in Lipovljani and also present in Bjelovar and Županja, suggesting localized transmission or persistence. Genotype B had the broadest geographic distribution, detected in Čakovec, Cerna, Mikanovci, Lipovljani, Županja, and Novoselec (Žutica), indicating either a more widespread reservoir or greater mobility across regions. Genotype D showed intermediate clustering, primarily in Lipovljani, with additional isolates from Bjelovar and Županja. Genotypes A and E were each confined to Mikanovci, supporting localized occurrence. Genotype F was restricted to Nova Subocka, while Genotype G was found only in Čakovec. These findings highlight a significant correlation between genotype structure and geographic origin, pointing to spatially distinct transmission patterns and region-specific reservoirs within the Croatian *Leptospira* population.

### 3.5. Phylogenetic Relationships of Croatian Isolates

**Figure 4:**
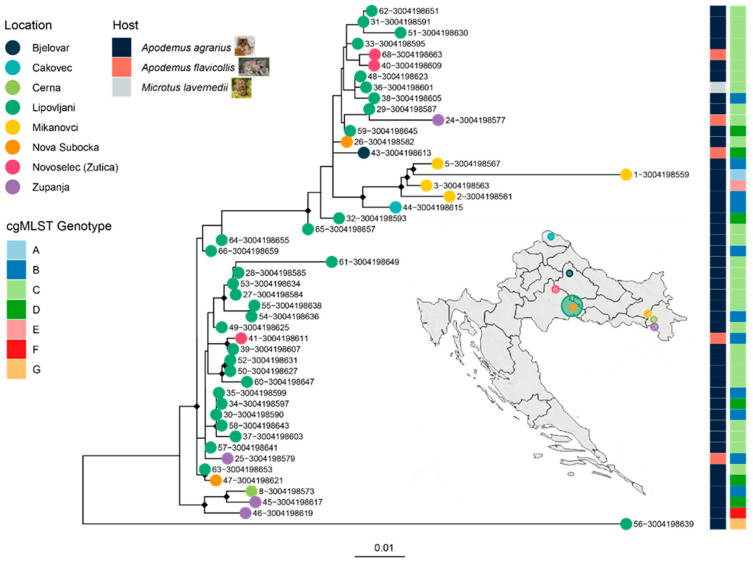
Maximum likelihood (ML) phylogeny of 48 *Leptospira kirschneri* isolates from Croatia, annotated by sampling location, host species, and cgMLST genotype. A maximum likelihood tree was constructed from a core SNP alignment of 48 *L. kirschneri* genomes collected from rodent hosts across multiple locations in Croatia. Tip colors correspond to geographic origin, as shown in the legend. The rightmost heatmaps indicate cgMLST genotype groups and host species (blue: *Apodemus agrarius*, red: *Apodemus flavicollis*, grey: *Microtus lavernedii*). Small rodent icons depict representative host species sampled. A map of Croatia (inset) highlights the sampling locations, with circle size proportional to the number of isolates from each site. Genotype groups defined by cgMLST are consistent with clade topology, supporting the presence of localized clonal expansions. The spatial clustering of strains and limited host diversity suggest restricted circulation within rodent reservoirs and geographic foci.

A ML phylogeny was reconstructed from the core SNP alignment of the 48 *Leptospira* spp. isolates collected from various locations across Croatia. The tree revealed several well-supported clades, with key nodes marked by black diamonds indicating ultrafast bootstrap support values exceeding 90% (Figure 4). These high-confidence branches suggest robust evolutionary relationships among the isolates. Clustering patterns were strongly associated with geographic origin, as denoted by colored tip circles corresponding to the sampling sites. Notably, isolates from Lipovljani and Mikanovci formed distinct monophyletic groups, reflecting localized evolutionary divergence.

The heatmap adjacent to the phylogeny annotated each isolate by host species and cgMLST genotype group (A–G), revealing tight concordance between phylogenetic structure and genotype. For instance, Genotype C isolates, predominantly from Lipovljani, clustered into a single, well-supported clade. Similarly, Genotype B showed some spatial dispersion but maintained phylogenetic cohesion.

### 3.6. Phylogenetic Placement of Croatian L. kirschneri Isolates in a Global Context

**Figure 5:**
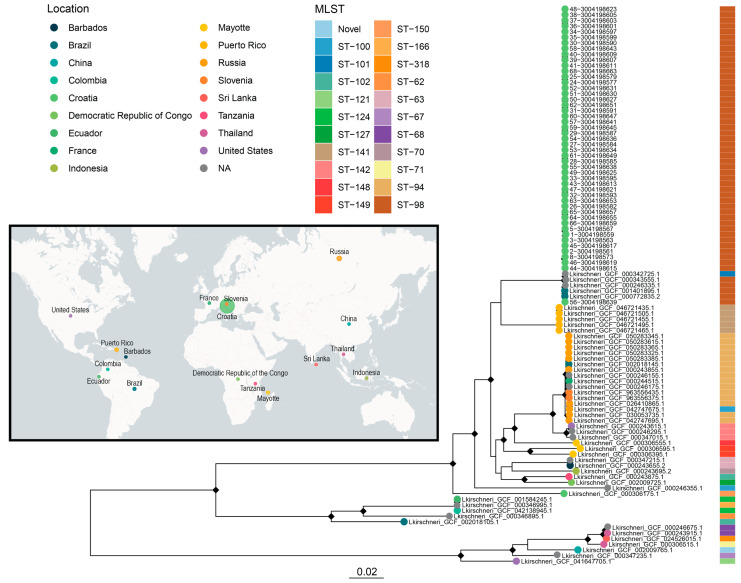
Maximum likelihood phylogeny of 99 *Leptospira kirschneri* genomes from Croatia and global sources, annotated by location, cgMLST genotype, host, and sequence type. A maximum likelihood tree was generated from a core SNP alignment of 99 *L. kirschneri* genomes, including 48 rodent-derived isolates from Croatia and 51 publicly available assemblies from the NCBI RefSeq database. Tips are colored by geographic origin (map and leftmost legend), with the accompanying sidebar denoting MLST sequence type. The NCBI RefSeq genomes originate from diverse global locations, spanning Africa, Asia, Europe, and the America, representing a wider range of MLST types, including ST-121, ST-124, ST-127, and others. The inset world map illustrates the geographic distribution of all the sampled isolates, highlighting the distinct separation between the Croatian and global *L. kirschneri* lineages, with circle size proportional to the number of isolates from each geographical location.

A ML phylogeny was constructed from a core SNP alignment of 99 *L. kirschneri* genomes, including 48 isolates from Croatia and 51 publicly available reference genomes from the NCBI RefSeq database (Figure 5). The resulting tree revealed a strongly supported monophyletic clade comprising all the Croatian isolates, indicating their close genetic relatedness and suggesting the presence of a regionally dominant lineage circulating in the country.

In contrast, the global reference genomes represented a diverse array of sequence types and geographic origins, spanning Brazil, China, Russia, the Democratic Republic of the Congo, Colombia, Puerto Rico, Ecuador, the United States, France, Slovenia, Thailand, Sri Lanka, Indonesia, Tanzania, Mayotte, and Barbados. These genomes encompassed multiple MLST sequence types, including ST-121, ST-124, ST-127, ST-141, and several novel STs, highlighting the global diversity of *L. kirschneri*. It is noteworthy that all the Croatian isolates were assigned to MLST ST-98, a sequence type shared with only a few global strains from Brazil and an unknown location. The same strains were also closely clustered in the phylogenetic analysis with the Croatian isolates, all of which had previously been identified as the *L. kirschneri* serovar Mozdok. This double concordance in sequence type and phylogenetic position supports the hypothesis of a geographically confined but globally dispersed lineage representing the *L. kirschneri* serogroup Pomona, likely the serovar Mozdok or Tsaratsovo, that has undergone long-term in situ evolution in Croatia, with limited evidence of international dissemination based on a few closely related global strains.

## 4. Discussion

Leptospirosis is one of the most common zoonotic infections of global importance but is still frequently underdiagnosed and neglected. In Croatia, leptospirosis is an important endemic zoonosis, occurring both in natural and increasingly in synanthropic foci. The unique combination of favorable climate and complex geomorphology of the country favors high species diversity, including a dense and diverse rodent population, which is the main reservoir of pathogenic *Leptospira* spp. [28,37]. These ecological conditions contribute to the endemic nature of the disease and maintain active transmission cycles in different geographical regions.

In previous studies, the serogroup Pomona has been consistently identified as the most widespread in both domestic and wild animals in Croatia using methods such as MAT, PFGE, MLST, and cgMLST [17,18,19,28,51]. Small rodents are considered important reservoirs responsible for the maintenance and spread of leptospires in these natural foci. Among them, the black-striped field mouse (*Apodemus agrarius*) has been established as the dominant reservoir host for the serogroup Pomona, while the yellow-necked field mouse (*Apodemus flavicollis*) is mainly associated with the serogroup Australis [28,37]. The composition of rodent species in this study was consistent with previous results, with *A. agrarius* accounting for 42 of the 48 isolates, further supporting the dominant role of *A. agrarius* as a reservoir for the serogroup Pomona serovar Mozdok in Croatia. Similar observations have been observed in other European countries, where the serovar Mozdok is maintained by small rodents [52,53]. While *A. agrarius* dominated overall in our study, isolates of *A. flavicollis* and *M. lavernedii* also formed distinct clusters, raising the possibility of limited host specificity or niche adaptation within the *L. kirschneri* strains. Whether these patterns reflect ecological constraints, selective pressures, or recent host jumps warrants further investigation.

Although the serogroup Pomona includes eight serovars distributed among five *Leptospira* species, the most frequently detected serovars in Croatia were Mozdok, Tsaratsovo, and Pomona [28,37,54]. Notably, Mozdok and Tsaratsovo belong to *L. kirschneri*, whereas Pomona belongs to *L. interrogans*. In this study, serological typing confirmed that all 48 isolates belong to the serogroup Pomona, confirming the dominant role of this serogroup in Croatia.

This study represents the first detailed whole-genome investigation of *Leptospira* isolates from Croatia and provides unprecedented resolution for species confirmation, strain-level comparison, and population structure analysis. The ANI analyses confirmed the species-level assignment of all 48 isolates to *L. kirschneri*, with bidirectional ANI values of 97–99%, both among the Croatian isolates and relative to the reference *L. kirschneri* genomes, while maintaining clear separation from other species. This indicates a highly clonal and distinct population circulating in Croatia.

Despite this overall clonality, cgMLST revealed seven genotype clusters, many of which were associated with specific geographic locations. These patterns were mirrored by SNP-based ML phylogenetic analyses, which revealed strong geographic structuring, particularly in regions such as Lipovljani and Mikanovci. This spatial structuring of cgMLST genotypes suggests localized transmission patterns and potential environmental or ecological segregation among the *L. kirschneri* populations in Croatia. The observed congruence between the cgMLST genotypes and phylogenetic clades suggests localized microevolution or ecological separation within the Croatian rodent reservoir system. Overall, these results suggest that *L. kirschneri* in Croatia exhibits structured genetic diversity driven by both geographic and host-related factors, with a strong phylogenetic signal supported by high bootstrap confidence. However, interpretation of geographic specificity should be made cautiously, given the limited availability of *L. kirschneri* genomes from outside Croatia for broader comparison.

While the cgMLST was useful for identifying broader genotype clusters, the minimum spanning tree did not fully recapitulate the fine-scale relationships observed in the SNP-based phylogeny. This discrepancy likely reflects the inherent difference in resolution between the two methods: cgMLST compares allelic variation across a fixed set of loci, collapsing all within-allele sequence variation, whereas SNP-based analysis captures single-nucleotide changes across the core genome. As a result, SNP phylogenies provide greater discriminatory power and more precise evolutionary inference, particularly among closely related isolates. The higher resolution of the SNP-based tree revealed distinct clustering within the cgMLST genotypes, emphasizing its suitability for inferring recent transmission events and microevolutionary patterns.

Phylogenetic clustering was consistent with both MLST designations and geographic origin, supporting the hypothesis that the Croatian L. *kirschneri* isolates form a regionally endemic lineage. A global comparison with publicly available *L. kirschneri* genomes confirmed that the Croatian isolates form a strongly supported monophyletic clade that is distinct from other global isolates in both SNP-based and ANI-based analyses. Only the Croatian isolates and a few global strains (from Brazil and an unknown location) were assigned to sequence type ST-98, indicating a limited global distribution and suggesting geographic confinement and possible long-term in situ evolution.

Ten isolates in this study were previously identified as the serovar Mozdok using PFGE [28], MLST, and monoclonal antibody tests. Since all 48 Croatian isolates belong to MLST ST-98 and are phylogenetically clustered with the *L. kirschneri* serovar Mozdok strains, it is plausible that they all represent the *L. kirschneri* serogroup Pomona serovar Mozdok, and less likely that they represent the serovar Tsaratsovo.

Interestingly, one isolate from the database, assigned to ST-101, clustered closely with the Croatian and the few global strains in the ST-98 group in the phylogenetic analysis. This ST-101 strain was previously identified as the *L. kirschneri* serogroup Pomona serovar Mozdok and caused pulmonary hemorrhagic lesions in an experimental hamster model, indicating high virulence [55]. This finding suggests the potential virulence of genetically related lineages within the serogroup Pomona. Moreover, other European studies further highlight the heterogeneous nature of the serogroup Pomona, indicating that both the *L. kirschneri* serovar Mozdok and the *L. interrogans* serovar Pomona can be associated with severe clinical manifestations in animals, underlining their pathogenic potential [52,53].

Additionally, these observations are in line with our previous clinical findings, where the serogroup Pomona has been frequently associated with severe disease manifestations, particularly leptospiral pulmonary hemorrhagic syndrome (LPHS) in dogs [56], although the specific serovars responsible within this serogroup remain unclear. Similar outcomes have also been reported in humans [57] and horses [58]. It is particularly relevant in our context, given the high incidence of leptospirosis in Croatia and the predominance of the serogroup Pomona in clinical cases in Croatia, but further studies are needed to clarify the role of individual serovars in the pathogenesis of LPHS. Altogether, these findings suggest a high pathogenic potential of the *Leptospira* spp. serogroup Pomona, highlighting the need for further investigation within a One Health framework to better understand its pathogenicity, host range, and environmental persistence.

Overall, these results emphasize the utility of whole genome sequencing to advance our understanding of *Leptospira* epidemiology. In addition to resolving species- and strain-level relationships, the pangenome analysis provided further insight into genomic cohesion and adaptive potential within this lineage, revealing a largely conserved core genome having limited accessory gene diversity. Traditional typing methods such as MLST and serology are unable to resolve this fine-scale population structure and genomic dynamics, underscoring the added value of comprehensive genomic approaches.

Our data suggest the presence of a stable, geographically structured, and potentially host-associated lineage of the *L. kirschneri* serogroup Pomona in Croatia, with limited gene flow from other global lineages, most likely corresponding to the serovar Mozdok, although the serovar Tsaratsovo cannot be ruled out.

The integration of ecological, serological, and genomic data in this study provides a comprehensive framework for understanding the population biology of the *Leptospira* spp. serogroup Pomona, probably one of the most pathogenic serogroups with high evolutionary propulsion in terms of pathogenicity, in the context of One Health. The genomic characteristics of the local strains allow comparative analysis with other sequenced pathogenic genomes of *Leptospira* spp. and thus help in the development of further diagnostic tests and vaccines. In addition, this work helps to gain genetic and epidemiological insights that improve knowledge about pathogenic infections with *Leptospira* spp.

Future studies should be extended to environmental and clinical isolates as well as additional hosts to elucidate transmission routes and the potential for cross-species infections. The identification of this cohesive local lineage underscores the importance of targeted surveillance and localized control strategies for leptospirosis in Croatia.

## Data Availability

Raw sequencing data for the 48 *L. kirschneri* isolates from Croatia generated in this study have been deposited in the NCBI Sequence Read Archive (SRA) under BioProject accession number PRJNA1279268. Assembled genomes and additional supporting data are available from the corresponding author upon reasonable request.

## Supplementary Materials

The following supporting information can be downloaded at https://www.mdpi.com/article/10.3390/pathogens14090860/s1. Figure S1: Presence–absence heatmap of pangenome gene content for 48 *Leptospira kirschneri* isolates from Croatia, with corresponding sampling location metadata.; Table S1: Panel of 14 reference hyperimmune sera used for serological typing of *Leptospira* spp. isolates, including corresponding serogroups, serovars, and reference strains.; Table S2: Metadata of *Leptospira* spp. isolates from small rodents: rodent species, sampling locations, collection dates, and associated SRA Information.; Table S3: Genome assembly quality and metrics for 48 Croatian *Leptospira kirschneri* isolates.; Table S4: Dataset of 118 genomes comprising 48 Croatian *Leptospira* isolates and 70 publicly available genomes representing all the recognized *Leptospira* type strains with whole-genome sequences available in the NCBI RefSeq database, used for the ANI analysis presented in Figure 1; Table S5: Dataset of 99 *Leptospira kirschneri* genomes comprising 48 Croatian isolates and 51 publicly available genomes from the NCBI RefSeq database used for the pairwise ANI analysis presented in Figure 2; Table S6: Multilocus sequence typing (MLST) and core genome MLST (cgMLST) profiles of 48 Croatian *Leptospira* spp. isolates.

## Abbreviations

The following abbreviations are used in this manuscript:

ANI	average nucleotide identity
BIGSdb	Bacterial Isolate Genome Sequence Database
cgMLST	core genome multilocus sequence typing
cgST	core genome sequence type
DNA	deoxyribonucleic acid
EDTA	ethylenediaminetetraacetic acid
GFF3	General Feature Format version 3
LPHS	leptospiral pulmonary hemorrhagic syndrome
MAT	microscopic agglutination test
ML	maximum likelihood
MLST	multilocus sequence typing
NCBI	National Center for Biotechnology Information
PBS	phosphate-buffered saline
PCR	polymerase chain reaction
PFGE	pulsed-field gel electrophoresis
RefSeq	Reference Sequence Database (NCBI)
SRA	Sequence Read Archive (NCBI)
SNP	single nucleotide polymorphism
ST	sequence type
WGS	whole genome sequencing
