# Supplementary material for: Whole Genome Characterization of Leptospira kirschneri Serogroup Pomona in Croatia: Insights into Its Diversity and Evolutionary Emergence
Source: Pathogens. 2025 Aug 29;14(9):860. doi: 10.3390/pathogens14090860 (PMC12472981; doi:10.3390/pathogens14090860)
Supplement: Supplementary file 1 [file pathogens-14-00860-s001.zip › Figure S1.pdf]

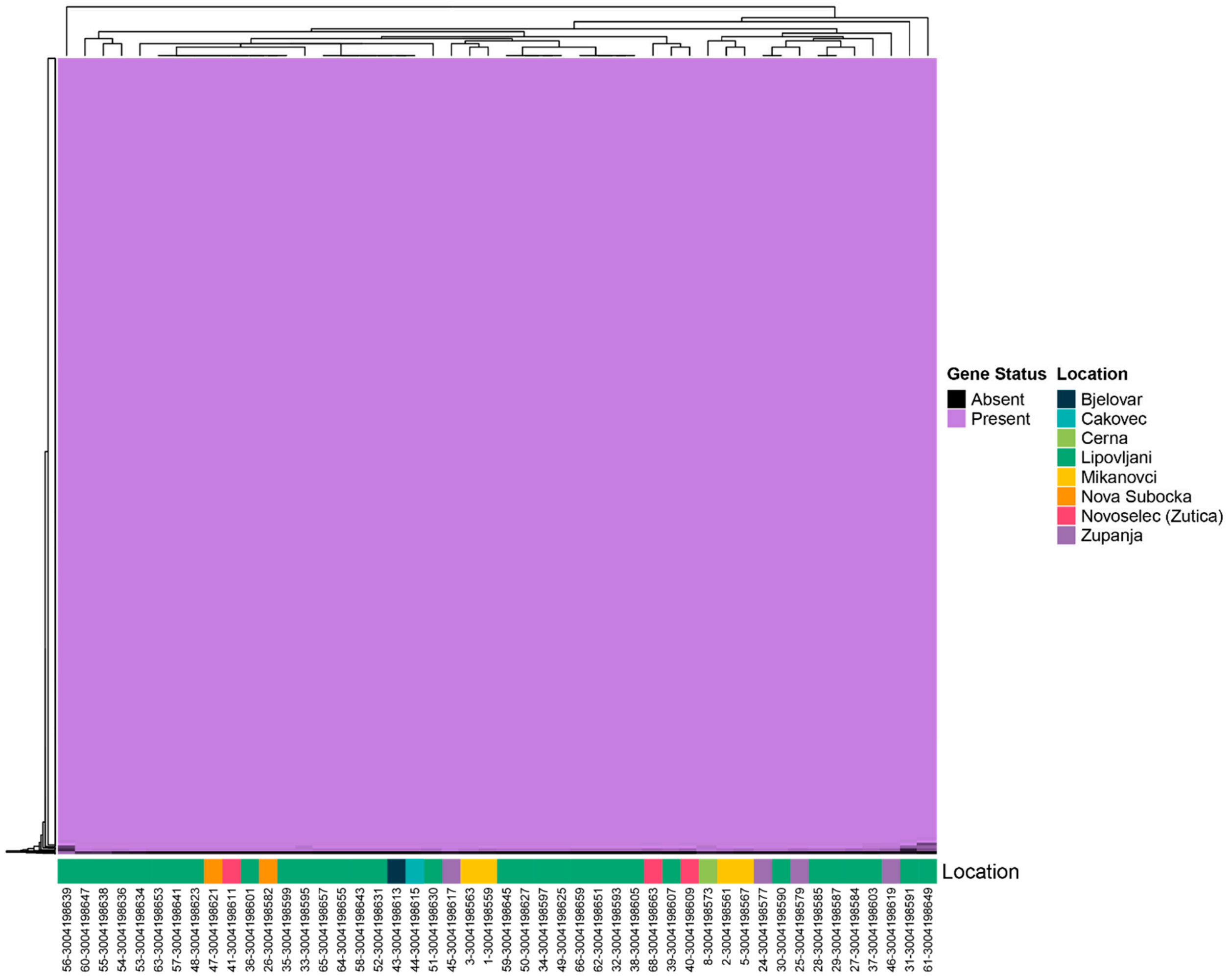

Supplementary Figure S1. Presence–absence heatmap of pangenome gene content for 48 *Leptospira kirschneri* isolates from Croatia, with corresponding sampling location metadata. Columns represent genome assemblies (isolates); rows represent gene clusters identified by Panaroo. Gene presence (purple) and absence (black) are indicated for each isolate. The dendrogram reflects hierarchical clustering based on gene content similarity. The adjacent heatmap annotates the sampling location of each isolate. Genome annotation was performed with Bakta, and pangenome analysis was conducted using Panaroo.
